# Molecular and genetic dissection of recursive splicing

**DOI:** 10.26508/lsa.202101063

**Published:** 2021-11-10

**Authors:** Brian Joseph, Chaz Scala, Shu Kondo, Eric C Lai

**Affiliations:** 1 Developmental Biology Program, Sloan Kettering Institute, New York, NY, USA; 2 Louis V Gerstner, Jr Graduate School of Biomedical Sciences, Memorial Sloan Kettering Cancer Center, New York, NY, USA; 3 Research Building 11F, Tokyo University of Science, Tokyo, Japan

## Abstract

Intronic ratchet points (RPs) are abundant within long introns in the *Drosophila* genome and consist of juxtaposed splice acceptor and splice donor (SD) sites. Although they appear to encompass zero-nucleotide exons, we recently clarified that intronic recursive splicing (RS) requires a cryptic exon at the RP (an RS-exon), which is subsequently always skipped and thus absent from mRNA. In addition, *Drosophila* encodes a smaller set of expressed exons bearing features of RS. Here, we investigate mechanisms that regulate the choice between RP and RS-exon SDs. First, analysis of *Drosophila* RP SD mutants demonstrates that SD competition suppresses inclusion of cryptic exons in endogenous contexts. Second, characterization of RS-exon reporters implicates exonic sequences as influencing choice of RS-exon usage. Using RS-exon swap and mutagenesis assays, we show exonic sequences can determine RS-exon inclusion. Finally, we provide evidence that splicing can suppress utilization of RP SDs to enable RS-exon expression. Overall, multiple factors can influence splicing of *Drosophila* RS-exons, which usually result in their complete suppression as zero-nucleotide RPs, but occasionally yield translated RS-exons.

## Abstract (precis)

Recursive splicing is an unusual form of stepwise mRNA processing involving exons with functional splice donors at their 5′ ends. We use molecular and genetic assays to show three parameters that influence mRNA isoform outcomes during recursive splicing in *Drosophila*.

## Introduction

Regulated and alternative splicing (AS) generates isoform diversity, yielding functional specialization and gene expression control (Fu & Ares, 2014; Ule & Blencowe, 2019). AS is critical to normal development and physiology, and consequently, splicing dysregulation can frequently lead to disease and cancer (Scotti & Swanson, 2016; Bonnal et al, 2020). Therefore, a more comprehensive understanding of mechanisms of splicing regulation are pertinent not only to enlarge our perspectives on how the transcriptome is appropriately deployed but can facilitate the interpretation of disease mechanisms and their rational therapy.

**Figure 1.**
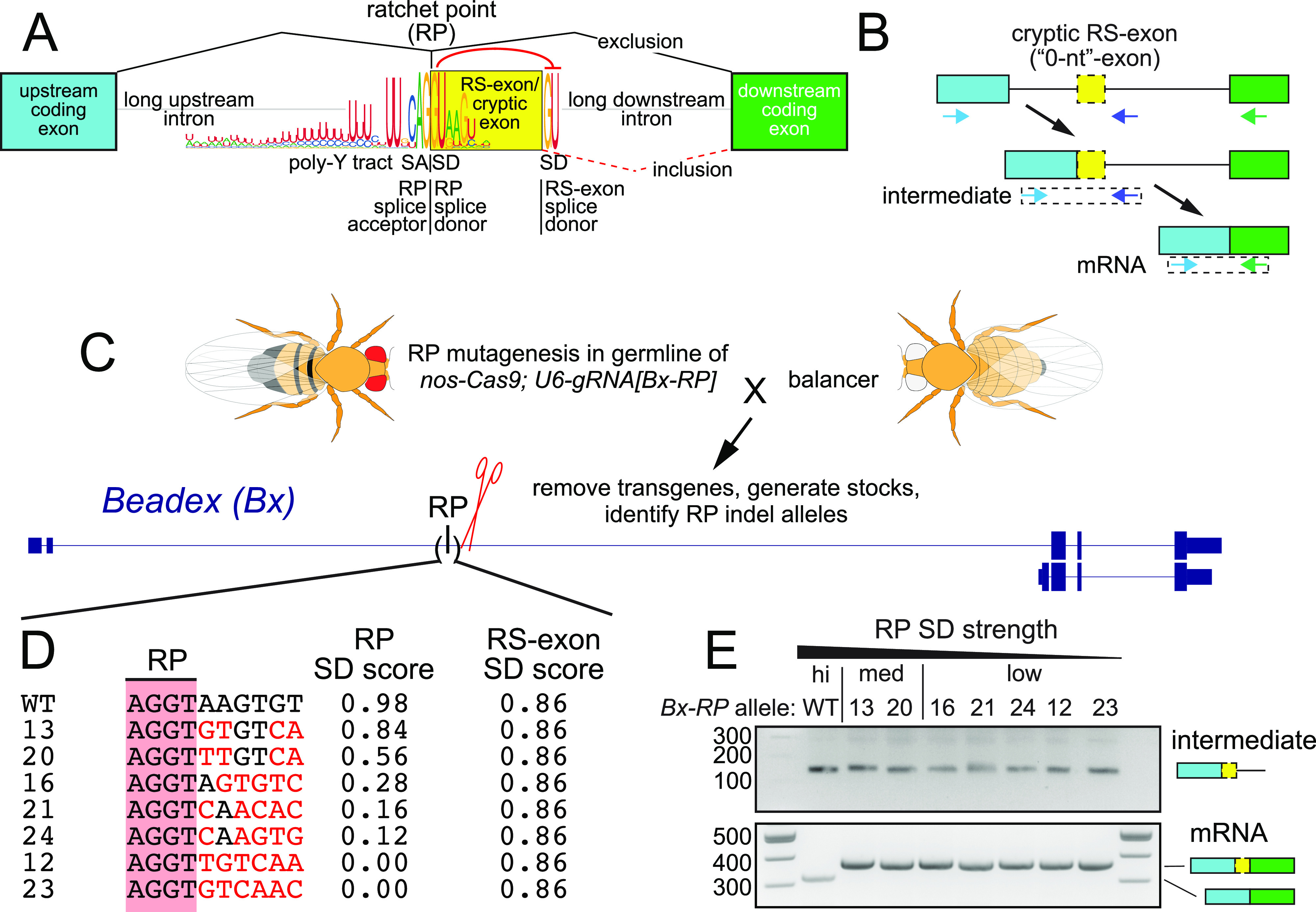
Recursive splicing (RS) and the impact of splice donor (SD) competition on cryptic exon exclusion. **(A)** Features of intronic RS in *Drosophila*. The ratchet point (RP) consists of a tandem splice acceptor and splice donor pair. There are hundreds of well-conserved RPs in *Drosophila*, which predominantly reside within long intronic contexts and exhibit the nucleotide preferences shown. The RP encompasses a cryptic RS-exon, which is short but of variable length (∼50 nt), and flanked by a downstream SD. In general, it is conceived that the RP SD suppresses the usage of the RS-exon SD by a competition mechanism because mutation of the RP SD results in inclusion of the cryptic RS-exon. **(B)** Proposed path for RS, with rt-PCR primers indicated that can monitor the recursive intermediate and mature mRNA product. **(C)** Transgenic CRISPR/Cas9 approach for efficient generation of RP mutants. *Bx* gene models displaying isoforms that use different transcription start sites. The RP is located within the longer isoform in the ∼31 kb intron 2. **(D)** CRISPR mutagenesis generated specific RP SD mutations as shown. Black nucleotides indicate matches to wild type, whereas red nucleotides designate changes relative in the *Bx*-RP SD. The allele ID is left of the sequence and changes to RP SD score on the right. The RS-exon SD score is also included; it is unchanged in these alleles. **(E)** Wildtype and RP SD mutants yield RS intermediate amplicons. However, unlike wild type, all weakened RP mutants include the cryptic RS-exon.

Recursive splicing (RS) constitutes a specialized class of splicing events and are defined by tandem 3′ splice acceptor (SA)-5′ splice donor (SD) pairs (Fig 1A). In *Drosophila*, these were originally characterized by the López laboratory at the *Ultrabithorax* (*Ubx*) locus. Its ∼73 kb intron harbors two 51 nt microexons (*m1* and *m2*), whose inclusion in a subset of *Ubx* isoforms is not generated by conventional AS, but instead by RS (Hatton et al, 1998). In this process, splicing at these short RS-exons regenerates a SD at the 5′ end of the RS-exon, also known as a ratchet point (RP) (Fig 1A). In the subsequent step, either the RP SD or the RS-exon SD can be used, yielding either exon skipping or exon inclusion, respectively.

Although these were originally thought to be rare cases, it was later recognized that many RPs exist within long *Drosophila* introns (Burnette et al, 2005; Duff et al, 2015). However, these were almost never found to be associated with expressed exons, even in deep and broad RNA-seq data, leading to the notion that they might reflect “0-nt” exons (Duff et al, 2015). Nevertheless, a characteristic “sawtooth” pattern observed in total RNA-seq data within introns provided clear evidence for splicing intermediates into RPs, even if these splice products are invisible in mRNA. More recently, we used experimental and computational strategies to reveal that intronic RPs are still associated with cryptic unannotated exons, which must be recognized before splicing (Joseph et al, 2018). Subsequently, the RP SD is predominantly used, instead of the cryptic RS-exon SD, resulting in removal of the cryptic RS-exon and the remaining intronic sequence (Fig 1B).

Such a model was demonstrated for regulatory control of RS-exons in mammalian genomes, which may potentially harbor >6,400 expressed RS-exons, whereas only nine fully suppressed RPs within long introns were noted definitively (Sibley et al, 2015; Blazquez et al, 2018; Boehm et al, 2018). In contrast, there are >500 intronic RPs in *Drosophila*, but <50 expressed RP-exons (Joseph et al, 2018; Pai et al, 2018). Moreover, RS of expressed RS-exons in *Drosophila* has largely only been inferred (Joseph et al, 2018), but validated only for *Ubx* (Hatton et al, 1998). Overall, the mechanism of RS across metazoans appears to be unified, but they differ in their general functional outputs between flies and mammals.

Cryptic exons at intronic RPs have curious properties. They bear ultraconserved sequences at the RP SA|SD motifs (Fig 1A) and where tested, are recognized constitutively. On the other hand, their exonic content is poorly conserved and generally out of frame. Therefore, it seems critical these cryptic RS-exons be recognized and then be excluded from mRNA (Fig 1B). Of course, canonical cassette exons are typically frame-preserving with lengths that are multiples of three (Long et al, 1995). Exceptions include so-called “poison exons,” in which exon utilization specifically yields an out-of-frame product that is down-regulated via nonsense mediated decay (Lareau et al, 2007; Carvill & Mefford, 2020; Thomas et al, 2020). For this class, poison exon usage represents a negative regulatory mechanism. However, there is a danger inherent in cryptic RS-exons, since they seem to be constitutively recognized. Thus, there can be severe consequences of accidental cryptic RS-exon inclusion in mRNA, especially those that lie within normal coding sequences, as these may alter translational reading frame or contain premature stop codons (Sibley et al, 2015; Joseph et al, 2018). For example, we documented that in vivo disruption of the intronic RP SD in two critical developmental regulators, *kuz* and *Ubx*, induces strong loss-of-function alleles that phenocopy classic mutants because of inclusion of frame-changing cryptic RS-exons (Joseph et al, 2018).

Implicit in these genetic observations is the notion that there must be strong mechanisms to promote skipping of cryptic RS-exons. Nevertheless, there must also be opposing regulatory forces, since at least some RS-exons are expressed as alternative mRNA isoforms (e.g., *Ubx*). However, little is known about the regulation of intronic RS-exon skipping. Thus far, the only indication about this has come from relative strengths of SDs at the RP versus RS-exon. In both insects and mammals, it appears that intronic RPs generally have stronger SDs than their corresponding RS-exons. Hence, SS competition was proposed as a basis for strong exon exclusion at RPs (“0-nt” splicing). Functional tests in cell culture using minigene reporters demonstrated that RS-exon inclusion can be regulated by modifying RP or RS-exon SD strength, providing support for splice site competition (Sibley et al, 2015; Joseph et al, 2018). Nevertheless, it remains unclear if the reduced introns of minigene reporters can appropriately mimic the challenges of long introns, or if splice site competition matters in the context of endogenous genes.

We may also infer that trans-acting factors may influence RS-exon inclusion, as is the case for other programs of alternative splicing regulation. With regard to *Ubx*, it was suggested that exonic sequences may enhance inclusion of the *Ubx-m1* RS-exon (Hatton et al, 1998). More recently, the Ule laboratory recognized that SDs of certain mammalian recursive splice sites are constitutively suppressed through the action of the core exon junction complex (EJC) and peripheral factor RNPS1. Accordingly, these RS-exons are typically included, but can be induced to be skipped under EJC loss-of-function conditions (Blazquez et al, 2018).

Here, we study the regulation of splice site selection within cryptic and expressed RS-exons. We consider the influence of SD strength, exonic elements, and upstream intron removal (as a proxy for EJC deposition). Our results suggest roles for all three in the contextual regulation of SD choice, providing new insights into the control of RS.

## Results

### RP mutagenesis of *Bx* shows that SD competition determines RS-exon inclusion

If the decision to include RS-exons was determined based on SD strength, with the RP SD outcompeting the RS-exon SD (Fig 1A), then weakening the RP SD should promote inclusion of RS-exons. This was tested by Ule and colleagues by transfecting a minigene RS reporter into mammalian cells (Sibley et al, 2015). Although their work supports the model for splice site competition, it is unclear if this mechanism is similarly determinant in the normal context of long host introns (e.g., 10s of kb), which are not convenient to manipulate or use in transient assays.

**Figure S1.**
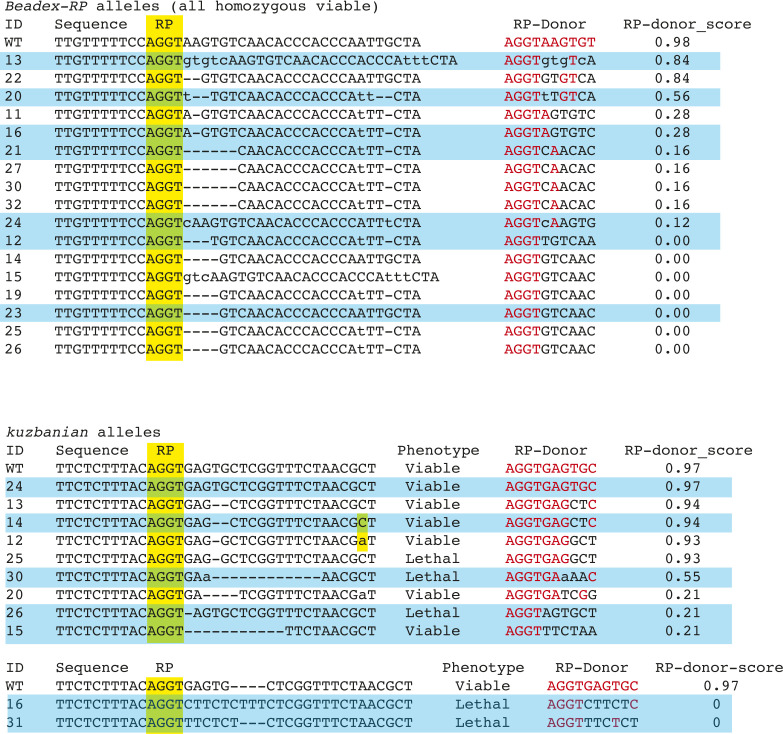
Isolation of ratchet point (RP) alleles at *Beadex* and *kuzbanian*. We used transgenic CRISPR-Cas9 to induce mutations near RPs of *Beadex* (*Bx*) and *kuzbanian* (*kuz*). Shown is a larger set of mutants obtained that preserve the core RP (juxtaposed splice acceptor and splice donor AG|GT dinucleotides) but bear downstream indels. The alignments on left are aligned to the reference sequence, whereas the RP-Donor columns at right depict the splice donor sequences that the spliceosome would encounter, along with their splice site scores as determined by NNSPLICE. All of the *Bx-RP* alleles are homozygous-viable (as the RP resides in the 5′ UTR), whereas some of the *kuz-RP* alleles are lethal, depending on whether they cause inclusion of frameshifting cryptic exon. Alleles highlighted in blue were saved and subjected to further molecular analysis; the others were since discarded owing to high similarity or identity of mutation patterns.

We decided to address this within true endogenous genomic contexts, using in vivo mutagenesis of *Drosophila*. We previously demonstrated feasibility for this approach by using transgenic CRISPR-Cas9 to mutagenize intronic RP SDs at several genes in the animal (Joseph et al, 2018). Here, we extended this effort with further screening to isolate additional RP mutant alleles at *Bx* (Fig 1C), yielding a broad panel of diverse *Bx[RP]* alleles (Fig S1). We were particularly interested in alleles that did not alter the core AGGT RP sequence, but instead resulted in deviations in positions +3 to +8 of the RP SD (Fig 1D). The *Bx* RS-exon resides in the 5′ UTR, and deletion of the RP SD in *Bx[∆RP]* is viable (Joseph et al, 2018); all of our new *Bx-RP* mutants were also homozygous viable. Analysis of splice site scores using NNSPLICE (Reese et al, 1997) showed that these mutant *Bx* alleles exhibit a range of RP SD strengths, from moderate (#13 and #20), weak (#16), to poor (#s 12, 21, 23, and 24). Importantly, all seven mutant RP SDs are predicted to be weaker than the cognate RS-exon SD, which remained unchanged (Fig 1D).

We used rt-PCR to assess molecular consequences of RP mutations on *Bx* processing. Because none of the induced mutations damaged the recursive SA (including the +1 to +2 position), we did not expect splicing into the cryptic exon to be altered. Indeed, analysis of an intermediate amplicon downstream of the cryptic exon (Fig 1B) yielded the expected products for all *Bx* mutants (Fig 1E). Therefore, the cryptic RS-exon was appropriately recognized in all cases. We then assessed RS-exon inclusion on mature *Bx* transcripts. Remarkably, rt-PCR of mRNA amplicons indicated that all changes to RP SD strength (moderate, weak or poor) resulted in a complete switch to RS-exon inclusion (Fig 1E). As all RP SD variants generated were weaker than the RS-exon SD, these data support a model in which SD strength drives alternative splicing. Thus, the functional output to include or exclude the *Bx* RS-exon in vivo correlates well with usage of the stronger SD (RP or cryptic) at the cryptic exon cassette.

### RP mutagenesis of *kuz* generalizes the impact of SD competition on “0-nt” splicing

**Figure 2.**
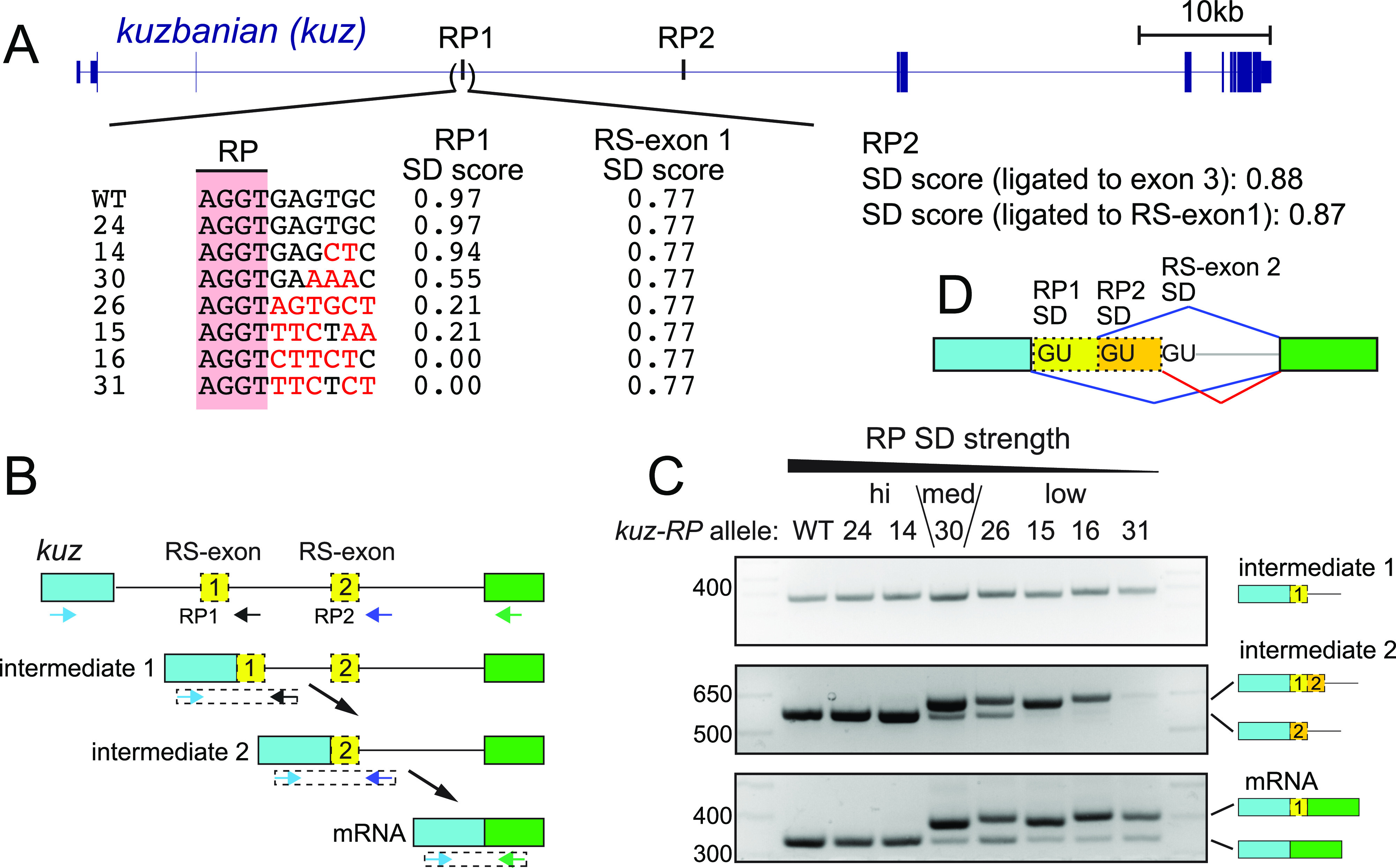
Weakened *kuz* RP1 splice donor (SD) causes recursive splicing (RS)-exon inclusion and enables analysis of downstream RS. **(A)** *kuz* gene models displaying two evenly spaced ratchet points (RPs) within its ∼50 kb intron 3. CRISPR/Cas9 was used to recover the RP1 SD mutations shown. Black nucleotides indicate matches to wild type, whereas red nucleotides designate changes relative in the *kuz*-RP SD. The allele ID is left of the sequence and changes to RP1 SD score on the right. The unaltered RS-exon SD score is also included for reference. **(B)** A model for *kuz* sequential RS. PCR amplicons are displayed using dotted boxes and primers as arrows. **(C)** Wild type and RP1 SD mutants yield similar RP1 intermediate amplicons. However, differences can be observed for RP2 intermediate and mRNA amplicons. Conversion of the high scoring RP1 SD to a medium or low scoring SD results in cryptic exon inclusion in RP2 intermediates and mRNA. Interestingly, whereas RP2 intermediates exhibit a steady conversion from cryptic exon skipping to fully cryptic exon inclusion as the RP1 SD weakens, mRNA amplicons always yield a minor level of cryptic exon skipped products (i.e., mature mRNAs). As *kuz* RS appears to be constitutive, the data suggest that weakened *kuz* RP1 SD can become activated to produce exon-skipped products (see Fig S2). **(D)** Multiple choices of SD to the downstream coding exon during *kuz* RS.

To broaden these results, we perturbed RS at *kuzbanian* (*kuz*). It follows similar principles of cryptic RS-exon suppression as *Bx* but is more complex because its long intron contains two RPs. We used CRISPR/Cas9 to obtain *Drosophila* strains bearing RP SD variants in the first RP (*kuz-RP1*). We characterized six mutants that preserve the core RP SD dinucleotide, but progressively weaken it from the optimal consensus (Figs 2A and S1). These included variant #14, which bears two nt substitutions at positions +6 and +7 of the SD and induces a slight decrease in splice score from 0.97 to 0.94 (1.00 being the highest). Another variant (#30) contained substitutions at additional positions, resulting in a moderate score (0.55). Finally, four mutants bear changes in positions +3 to +8, yielding very weak SD scores in the 0–0.21 range (Fig 2A). An allele lacking mutations in the RP SD (#24) was used as an additional control. Similar to the *Bx* RP-mutant series, the *kuz* RS-exon SD was unchanged in all mutants. Critically, only #24 (control) and #14 had RP SDs that were significantly stronger than the RS-exon SD (Fig 2A).

We again used rt-PCR to assay the consequences of mutating the *kuz* RP1 SD. We earlier showed, using analogous in vivo core RP SD disruption alleles, that RS in *Drosophila* is constitutive (Fig 1B) (Joseph et al, 2018). Therefore, *kuz* intron 3 is not removed in one step, and instead processed as three smaller fragments using two RPs (Fig 2B). We first examined the two obligate splicing intermediates that arise from activation of RP1 and RP2 (Fig 2B). The first intermediate, which indicates processing of *kuz* RP1 (and mutant RP1), was unaffected by mutations to the SD (Fig 2C, intermediate 1). However, the second intermediate amplicon (indicating processing of *kuz* RP2) yielded an additional band from samples that had moderate to poor RP1 SD scores (Fig 2C, intermediate 2). The additional product was longer than expected and confirmed by Sanger sequencing to include RS-exon 1 – a clear indication of switching from usage of the SD from RP1 to RS-exon 1. The differences in the sizes of these bands correspond to distinct insertion or deletions present across the panel of alleles, as noted in Fig S1. We observed that inclusion of RS exon 1 in the second intermediate amplicon increased with stepwise decreases in RP1 SD strengths, and only began once the RP1 SD was significantly weaker than the RS-exon 1 SD (Fig 2A–C, intermediate 2, “low” strength lanes). Together, these results provide further strong support to RP SD strength as a major determinant of RS-exon inclusion.

We also examined the molecular consequences of RP1 SD mutations on *kuz* mRNA. Here, we sought to understand the conversion of the second intermediate into mRNA. In wild type, the RP2 SD outcompetes the RS-exon 2 SD, generating mRNA that skips RS-exon 2 (Fig 2B). We confirm this via sole accumulation of the fully processed *kuz* mRNA amplicon in wild type, as well as RP mutants #24 (control) and #14, which retain strong RP1 SD and yield only canonical second intermediate (Fig 2C, mRNA, “hi” strength lanes). However, because mutants with moderate (#30) to poor (alleles #26, 15, 16, 31) RP1 SD include RS-exon 1 in the second intermediate (Fig 2C, intermediate 2), we wondered how this would affect downstream intron removal. We emphasize that this regulatory situation has not previously been modeled accurately using minigenes.

**Figure S2.**
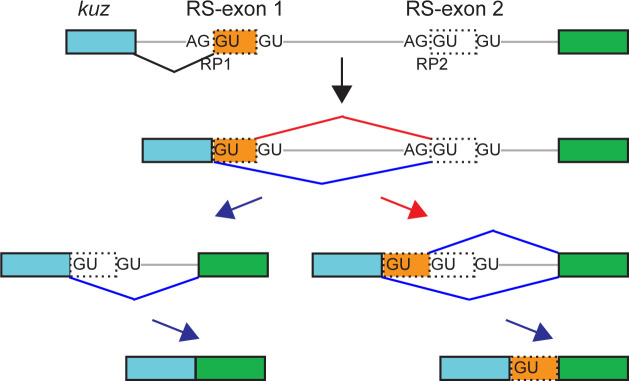
Proposed intron removal trajectories for *kuz* RP2 intermediate and mRNA products from ratchet point (RP) splice donor (SD) mutants. The schematic depicts the utilization of SD sequences that yield the observed pre-mRNA intermediates and mRNA in rt-PCR tests of the panel of *kuz-RP* mutants. Usage of RP SD is indicated with dark blue edges and arrows, whereas usage of the recursive splicing (RS)-exon SD is indicated in red. When *kuz* RP1 SD is mutated to a poor splice site, the RS-exon SD is activated (red) and the RP2 intermediate includes the cryptic RS-exon 1 (dotted orange box). However, in the next step (conversion to mRNA), one of the two remaining RP SD is used to generate canonical mRNA or mRNA with cryptic RS-exon 1 retention. Surprisingly, weak and poor RP1 SD can become selected for usage at the RP2 intermediate stage, despite the presence of two other strong SD.

Because these intermediates will contain three SDs (RP1 SD, RP2 SD, and RS-exon 2 SD, Fig 2D), we hypothesized that the strongest SD would be used dominantly. Of the three, RP2 SD is stronger than either RS-exon 2 SD or mutant RP1 SD. This prediction was supported by rt-PCR tests that showed RS-exon 1 inclusion in mRNA (Fig 2C, mRNA, lanes 4–8). Surprisingly though, whereas mutants 15, 16, and 31 only produced second intermediates that fully included RS-exon-1, a fraction of these are converted into RS-exon-1–skipped mRNAs (Fig 2C, mRNA, asterisks). This suggests that the significantly weaker RP1 SD can also become used during conversion to mRNA (Fig S2) and hints that other factors may also regulate RS-exon inclusion.

Overall, these tests constitute the first in vivo evidence that SD strength is a potent determinant of RS exon inclusion or skipping in the endogenous setting. Furthermore, as most *Drosophila* RPs tend to have strong regenerated SD, this is consistent with the end result that most of their cryptic RS-exons are skipped.

### Cryptic RS- and RS-exon reporters exhibit a wide range of alternative splicing patterns

**Figure 3.**
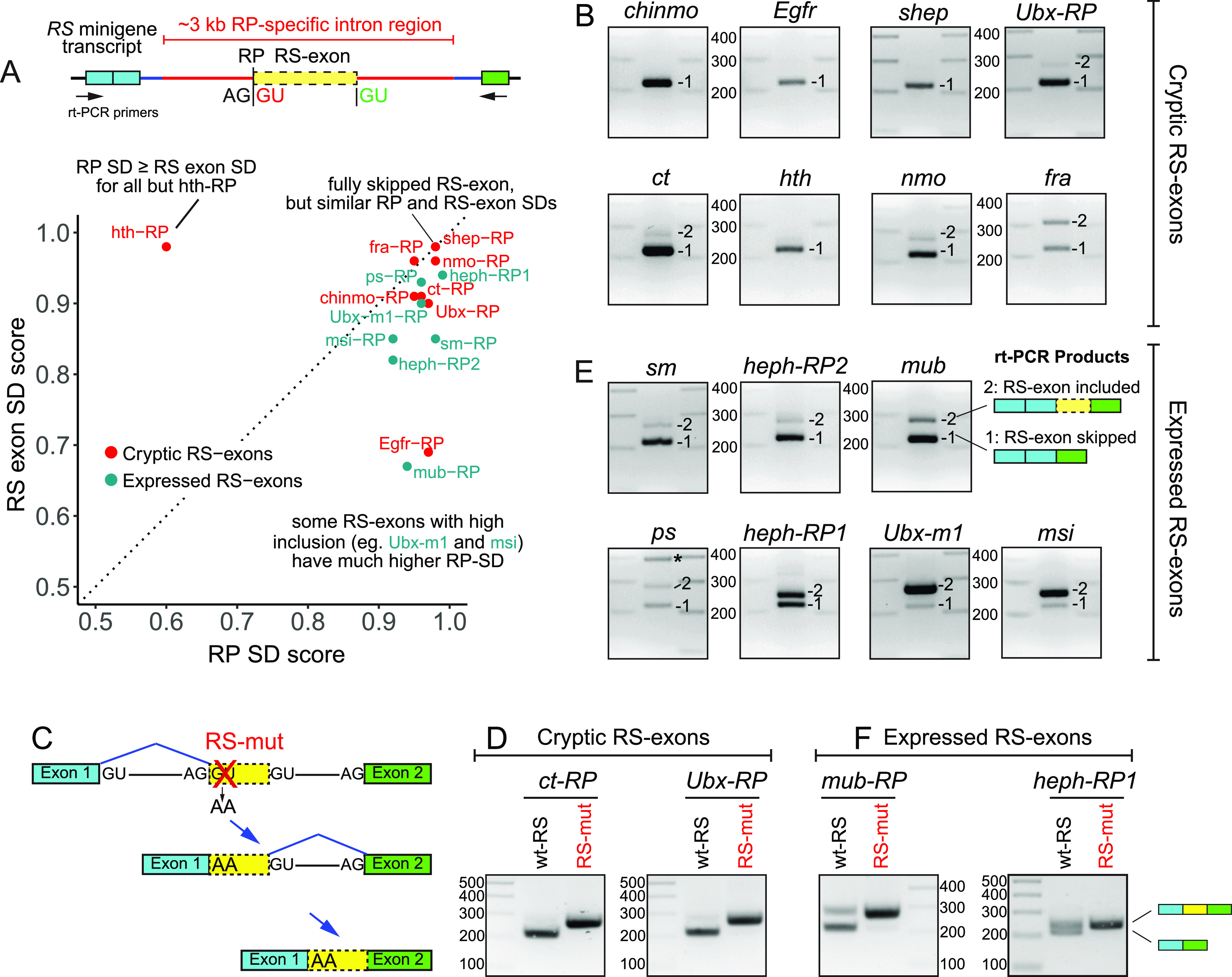
Systematic analysis of *Drosophila* recursive splicing (RS)-exon splicing properties. **(A)** Above: Test backbone for RS splicing minigene reporters. We cloned ∼3 kb centered on the ratchet point (RP) and RS-exon from each test locus (in red) into a splicing minigene bearing the flanking exonic/intronic context of *kuz-RP1*. Common rt-PCR primers are used to evaluate the inclusion or exclusion of the RS-exon. Below: Comparison of RP and RS-exon splice donor (SD) scores using NNSPLICE. Selected recursively spliced loci whose inclusion/exclusion patterns are not well-explained by SD competition are indicated. **(B)** rt-PCR of splicing reporters containing cryptic RS-exons. For most substrates, the expected exon skipped amplicon was the major product. **(C)** Strategy to validate RS in minigene splicing reporters. Schematic of the RS pathway after RP SD disruption. Critically, the skipped cryptic RS-exon will be converted to constitutively included after this mutation. **(D)** RP SD mutations in cryptic RS-exon substrates lead to complete inclusion of the RS-exon in mRNA. **(E)** rt-PCR of splicing reporters containing expressed RS-exons. A range of RS-exon inclusion levels can be observed for these RS substrates. Notably, some do not match expectations based on SD scores (see panel 3A). For instance, *msi* and *Ubx-m1* are dominantly included, despite having weaker RS-exon SD than their respective RP SD. **(F)** Validation that expressed RS-exons undergo RS because mutation of their RP-SDs yields constitutive exon inclusion.

Generating and assaying RS mutants in live animals provided valuable insights, but was laborious. We conducted further tests using minigene RS reporters. These contain constant flanking exon and adjacent intronic contexts from the recursively spliced *kuz* region, into which we place test RS regions and assay their processing in S2 cells (Joseph et al, 2018). We sought to identify other features that regulate RS-exon alternative splicing, for which we needed RPs that permitted differential inclusion of RS-exons. Accordingly, we assayed eight other cryptic RS-exons and seven expressed RS-exons into the splicing backbone (Fig 3A). To mimic the normal context of these RS regions, we cloned ∼3 kb centered on each RS-exon (Fig 3A). The RS-exon and RP SDs of these loci are plotted in Fig 3A.

Expression of cryptic RS-exon reporters predominantly yielded products in which the RS-exon was skipped (Fig 3B). For *chinmo*, *Egfr*, *shep*, *Ubx*, *ct*, and *nmo*, all of these had stronger RP SD than RS-exon SD (Fig 3A), consistent with the results from the in vivo mutagenesis tests. Interestingly, the *homothorax (hth)* RS reporter also yielded an exon skipped product (Fig 3B), despite having a substantially weaker RP SD than RS-exon SD (Fig 3A). In four of eight instances (*ct*, *Ubx*, *nmo*, and *fra*), we detected RS-inclusion in addition to the skipped amplicon. Although the levels of RS-exon inclusion vary based on reporter, they do not appear to correlate well with SD strengths (Fig 3A and B).

In theory, the RS-exon skipped products could be obtained through exon skipping, as opposed to RS. To account for this possibility, we generated mutant versions of two RS reporters (*ct-RP* and *Ubx-RP*) in which the RP SD were disrupted (Fig 3C). Under conditions of exon skipping, such mutations should not alter the reporter products. However, if spliced via RS, the mutant reporter should exhibit constitutive inclusion of the RS-exon (Fig 3C). Indeed, both mutant reporters fully switched from exon skipping to exon inclusion (Fig 3D). This extends our prior evidence (Joseph et al, 2018) that cryptic RS-exon reporters yield skipped products via RS.

Next, we examined the products of expressed RS-exon reporters (Fig 3A). For these, rt-PCR products revealed variable proportions of RS-exon inclusion and skipped amplicons (Fig 3E). For *sm*, *heph-RP2* and *mub*, the dominant amplicon was the exon-skipped product, whereas *Ubx-m1* and *msi* yielded mostly the exon included product. The remainder, reporters of *ps and heph* (RP1), produced equal proportions of skipped and included amplicons (Fig 3E). Importantly, the predicted RS-exon SD was used in all cases with RS-exon inclusion, the only exception being the *pasilla (ps)* reporter, which in addition to the predicted RS-exon SD, also activated another cryptic exon at the boundary of the *kuz* and *ps* intronic sequences (Fig 3E, asterisk). As with the cryptic RS-exon reporters, we used RP mutagenesis to verify that RS was the basis for the observed alternative splicing patterns (Fig 3F). Notably, comparison of SD strengths revealed that seven of seven reporters in this category have stronger RP SD (Fig 3A). The *Ubx* (m1) and *msi* reporters were particularly noteworthy as these mostly yield exon inclusion isoforms, despite having stronger RP than RS-exon SDs. Overall, this broad survey of RS reporters indicates that mechanisms other than SD competition are likely to regulate inclusion of RS-exons.

### Exonic elements can determine RS-exon alternative splicing

**Figure S3.**
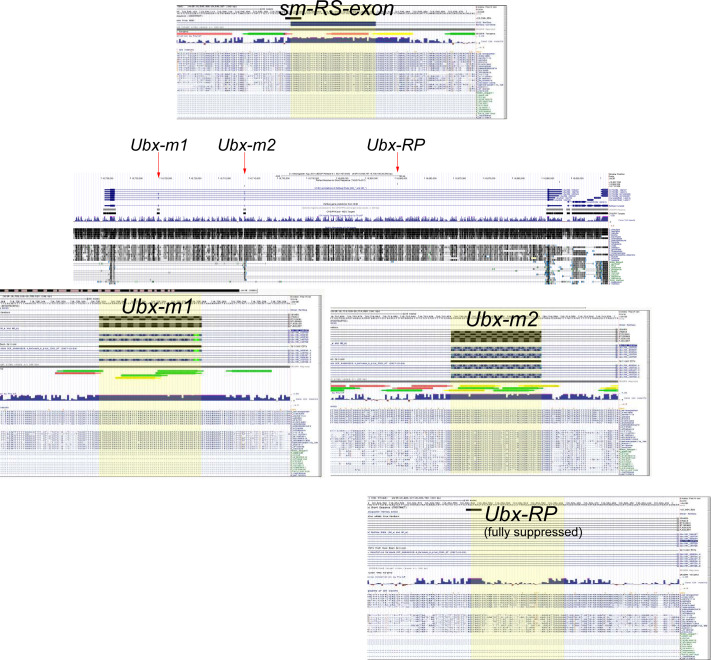
High conservation of expressed recursive splicing (RS)-exons. Shown are UCSC genome browser screenshots for expressed RS-exons from *smooth* (*sm*) and *Ultrabithorax* (*Ubx*). Note that *Ubx* contains two expressed RS-exons (m1 and m2) and one “0-nt” ratchet point that contains a cryptic non-translated exon.

Because RS reporters differing in the content of intronic RS sequence yield highly variable processing, we tested the possibility that these could be the effects of splicing regulatory elements (SREs) found within the reporter. As the introns flanking RPs are typically large, we first interrogated the contribution of RS-exon content. SREs are typically found within exons, or proximal to exons within introns (Fu & Ares, 2014; Ule & Blencowe, 2019), and recognition of constitutive exons may be aided by exonic splicing enhancers (ESEs) (Wang & Burge, 2008). Therefore, we first examined RS-exons for their conservation patterns. Most cryptic RS-exons are poorly conserved, but expressed RS-exons with coding potential can be conserved. Of our validated expressed RS-exon reporters, the *Ubx* microexon 1 (m1) and the RS-exon from *smooth (sm)* are deeply conserved across the Drosophilid phylogeny (Fig S3). However, as the *sm* RS-exon is not abundantly included in S2 cells (Fig 3E), we focused attention on the *Ubx-m1* reporter and the companion *Ubx-RP* reporter (Fig 3B and E).

**Figure 4.**
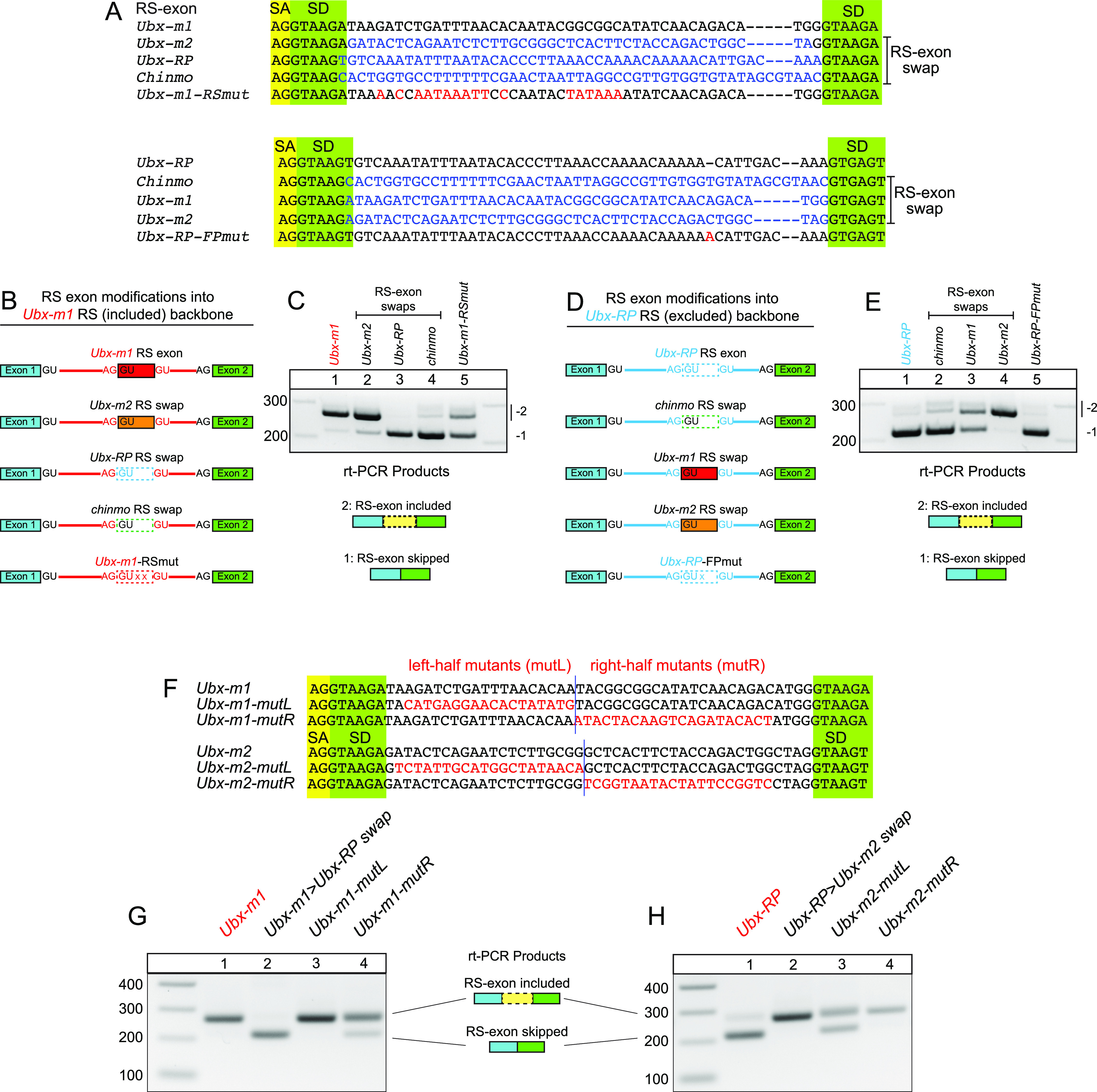
Recursive splicing (RS)-exon sequences can autonomously determine their inclusion. **(A)** Sequences of the different wildtype, swapped and mutant RS-exons tested. Wildtype sequences are in black, RS-exons swaps are in blue and mutations are in red. **(B)** Schematics of RS-exon variants built on the *Ubx-m1* reporter. *Ubx-m1* specific intronic sequence in red. Only the 51 nt *Ubx-m1* RS-exon portion of the reporter was swapped with the RS-exons of *Ubx-m2*, *Ubx-RP*, or *chinmo*; *Ubx-m1-RS-mut* bears mutations internal to the RS-exon. **(C)** RS-exons contain information to regulate alternative splicing of RS-exons. The *Ubx-m1* RS-exon reporter is dominantly included. Swapping the *Ubx-m1* RS-exon with others mimics their inclusion or skipping behaviors. Moreover, the *Ubx-m1-RS-mut* reporter exhibits substantial skipping indicating that it is no longer appropriately included. **(D)** Schematics of RS-exon variants built on the *Ubx-RP* RS-exon reporter. *Ubx-RP*–specific intronic sequence in blue. Only the *Ubx-RP* RS-exon portion of the reporter was swapped with the RS-exons of *Ubx-m2*, *Ubx-m1* or *chinmo*. The *Ubx-RP-FP* variant converts this RS-exon to a frame preserving (FP) length. **(E)** The *Ubx-RP* RS-exon reporter is predominantly skipped. Swapping the *Ubx-RP* RS-exon with others mimics their inclusion or skipping properties. The *Ubx-RP-FP* reporter is largely exon-skipped, indicating that mRNA stability is not a major confounding factor. **(F)** Sequences of mutant variants of *Ubx-m1* and *Ubx-m2* RS-exons. **(G)** Mutation tests of the *Ubx-m1* RS-exon, which is dominantly included. As a control, swapping of its RS-exon with the *Ubx-RP* exon results in skipping. Mutation of the left half of *Ubx-m1* (mutL) did not affect processing, but mutation of its right half (mutR) resulted in substantial RS-exon skipping. **(H)** Mutation tests of the *Ubx-m2* RS-exon, which is dominantly included, even when inserted into the *Ubx-RP* backbone. The mutL variant was now substantially skipped, whereas the mutR variant exhibited normal inclusion.

All 51 nt of the *Ubx-m1* exon are ultraconserved across Drosophilid species, including the wobble positions of all 17 codons (Fig S3). This suggests that information beyond coding potential is under strong selection (Bomze & Lopez, 1994). To test if the *Ubx-m1* RS-exon contains relevant splicing determinants, we conducted both swap and mutagenesis experiments (Fig 4A). We first made precise replacements of the 51 nt *Ubx-m1* RS-exon, within the context of the *Ubx-m1* 3 kb intronic reporter, with other RS-exons with distinctive splicing behaviors. To this end, we tested another expressed RS-exon (*Ubx*-*m2*) and others that were fully excluded (*Ubx-RP* and *chinmo* cryptic RS-exons) (Fig 4B). These swaps do not modify sequence of the *Ubx-m1* RS-exon SD that lies immediately adjacent in the downstream intron (Fig 4A).

Remarkably, the modified reporters behaved in accordance with the RS-exon swap. For example, the *Ubx-m2* RS-exon swap yielded predominantly exon inclusion (Fig 4C, lane 2). In stark contrast, the *Ubx-RP* and *chinmo* RS-exon swaps produced exon skipping (Fig 4C, lanes 3 and 4). Because all RS-swaps maintained the stronger RP SD (Fig 3A), these results argue that elements within the RS-exon are additional determinants of RS alternative splicing.

We reciprocally tested whether we could convert a skipped RS-exon reporter (i.e., “0-nt” RP splicing) into an expressed exon format. To test this, we used the *Ubx-RP* reporter, which is predominantly skipped (Fig 3B). We again replaced its cryptic RS-exon with the same panel of RS-exons (Fig 4A and D). Once again, the modified RS reporters reflected autonomous behaviors of the swapped RS-exons. Whereas *chinmo* RS-exon was mostly skipped, *Ubx-m1* produced a switch to an even proportion of both products. Meanwhile, the *Ubx-m2* swap yielded a complete switch to exon included (Fig 4E, lanes 1–4). We note that a longer, minor product can be observed for both reporters (Fig 4E) because of unexpected activation of weak SD downstream of the annotated RS-exon.

As noted, cryptic exons residing at intronic RPs are inevitably skipped, and have strong propensity to be out of frame. We therefore considered the possibility that reading frame might somehow influence the accumulation of spliced products, which we have measured only in steady state. The *Ubx-m1* and *Ubx*-*m2* RS-exons are frame preserving (51 nt, each), whereas the *Ubx-RP* and *chinmo* RS-exons are not (53 and 56 nt). To assess this possibility, we modified the *Ubx-RP* reporter to make the RS-exon frame preserving (Fig 4A, RP-FP-mut – 54 nt). However, this reporter was still fully skipped (Fig 4E).

In the swap tests above, the exon terminal sequences were exchanged, which might in principle affect recognition by spliceosome components. To test more rigorously if internal RS-exon sequences can influence splicing, we conducted further mutagenesis of both *Ubx* expressed RS-exons. We first mutated a number of positions within the *Ubx-m1* RS-exon that is normally included, without affecting the recursive SD (Fig 4A, m1-RS-mut). These alterations substantially converted the *Ubx-m1* RS-exon reporter to an exon skipping profile (Fig 4C, lanes 1 versus 5). We also created variants in which the left or right halves of the *Ubx-m1* RS-exon were scrambled, whilst retaining the original RS-exon termini (Fig 4F). We observed that mutation of the right-hand portion of *Ubx-m1* resulted in substantial skipping of the RS exon (Fig 4G). We also conducted similar mutagenesis of the *Ubx-m2* RS-exon swap into the *Ubx-RP* backbone, where *Ubx-m2* sequences autonomously determine RS-exon inclusion (Fig 4D and E, lane 4). Here, we found that mutation of the right half of the RS-exon was compatible with normal behavior, whereas alteration of the left half (without affecting the RP-SD) strongly compromised inclusion (Fig 4F and H). Thus, beyond the phenomenon of SD competition, internal exonic sequences can determine the outcome of RS-exon alternative splicing.

The ultraconserved nature of *Ubx-m1* and *Ubx-m2* exons (Fig S3) indicates that they contribute conserved peptides to Ubx protein isoforms, but also hints at the possibility of regulatory information beyond coding status. Our experiments show that although reading frame does not seem to influence RS splicing, internal exonic sequences can strongly influence RS-exon inclusion independently of SD competition. These data are consistent with the notion that trans-acting regulators may recognize these particular RS-exons to promote their inclusion.

### Splicing may stimulate RS-exon inclusion

The EJC is deposited ∼20–24 nt upstream of exon junctions during the splicing reaction (Schlautmann & Gehring, 2020). If RS is similar to canonical splicing, removal of the upstream intron fragment should deposit the EJC ∼20–24 nt upstream of the RP SD. Thus, it is reasonable to consider if the EJC may regulate RS. Two sources of evidence suggest this is plausible. First, the EJC is needed for accurate processing of long introns (Ashton-Beaucage et al, 2010; Roignant & Treisman, 2010), and otherwise regulates splice site activation at specific loci (Hayashi et al, 2014; Malone et al, 2014). Second, the EJC suppresses RS on constitutive exons, to promote RS-exon inclusion in both mammals (Blazquez et al, 2018; Boehm et al, 2018) and *Drosophila* (Joseph & Lai, 2021). Therefore, we sought to examine how splicing, implicitly via the EJC, may influence *Drosophila* intronic RS.

**Figure 5.**
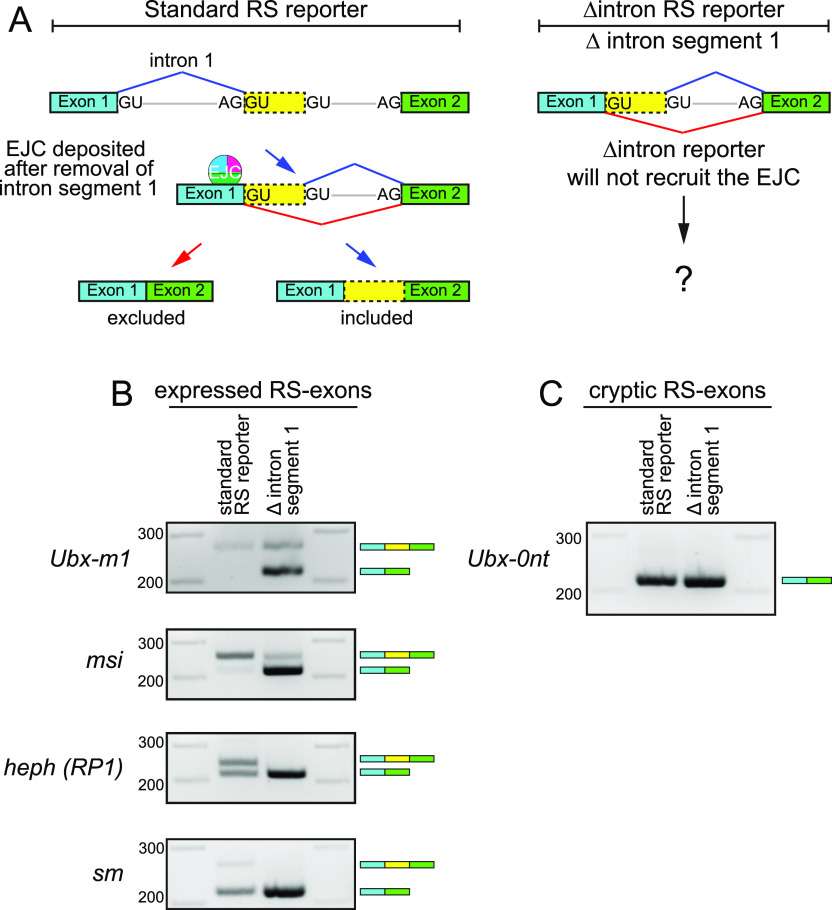
Intron pre-removal, as a proxy of exon junction complex (EJC) loss, induces recursive splicing (RS)-exon skipping. **(A)** Left: model of RS-exon splicing, including the deposition of the EJC after removal of intron segment 1. Right: schematic of ∆intron RS reporters. These will not recruit EJC prior to removal of intron segment 2. **(B)** ∆intron reporters for expressed RS-exons exhibit higher levels of exon skipping. **(C)** Cryptic RS-exon reporter from *Ubx-RP* that is normally skipped is unaffected by pre-removal of intron segment 1.

We selected four reporters that yielded a range of RS-exon inclusion, from low to high. To model the selective loss of EJC recruitment on these reporters, we deleted the upstream intron segment 1 (Fig 5A, ∆intron segment 1). Deletion of the intron segment mimics the RS-intermediate pre-mRNA without actually undergoing the splicing reaction, so these reporters are not expected to recruit the EJC. All four deletion constructs displayed an overall increase in RS-exon skipping (Fig 5B). The *Ubx-m1* and *msi* RS reporters, which are normally included, yielded predominantly skipped products when the upstream intron was removed (Fig 5B). More strikingly, the *sm* and *heph* ∆intron reporters yielded solely the exon-skipped amplicon.

To examine if splicing regulates cryptic RS-exons, we examined a *Ubx-RP*∆intron variant. Deletion of the upstream intron in this reporter had no discernible effects relative to the unmodified construct, both of which remained entirely skipped (Fig 5C).

These tests demonstrate that upstream intron splicing can impact the outcome of RS-exon splicing. Given the literature and mechanistic impact of deleting the upstream intron, we asked if EJC depletion alters RS-exon expression using EJC-RNAi datasets from S2 cells (Akhtar et al, 2019). However, we did not observe substantial or directional effects (Table S1). We note that a caveat of these tests is that a number of the best-expressed RS-exons are not detected in S2 cells; for example, cultured cells do not express *Ubx* (https://flybase.org/reports/FBgn0003944) (Cherbas et al, 2011). Thus, we could only examine 10 expressed RS-exons, even when using a generous cutoff (see the Materials and Methods section). Accordingly, these data do not definitively rule out involvement of the EJC in RS-exon splicing. Alternatively, the strong influence of SD competition (Figs 1 and 2) may be a sufficient determinant in these settings.

Table S1 Expressed recursive splicing-exon levels in EJC-depleted cells.

Overall, a favorable hypothesis is that the EJC may regulate RS-exon inclusion, although further study is needed. Nevertheless, these results indicate that upstream splicing is a positive factor for alternative splicing of *Drosophila* expressed RS-exons.

## Discussion

### Multiple factors influence choice between RP SD and RS-exon SD

Several factors are known to regulate splice site choices that underlie alternative splicing. These include *cis*-elements, *trans*-acting factors, the histone code, RNA modifications, RNAPII regulation, gene architecture and other factors (De Conti et al, 2013; Lee & Rio, 2015). Yet, despite the two decades that have passed since the first discovery of recursive splice sites in introns, relatively little is known regarding the mechanism of RS. In this study, we examine the roles of SD strength, exonic sequences, and the EJC, in influencing RS-exon inclusion. We apply the first set of broad in vivo mutagenesis of RPs in multiple genes in the animal, to show that progressively decreasing RP SD strength can convert cryptic RS-exons in expressed RS-exons. These data provide strong support for the SD competition model (Sibley et al, 2015), now using the endogenous setting and normal long flanking intronic contexts.

Reciprocally, we screen a substantial panel of RS minigene reporters to provide evidence that relative SD strength is insufficient to fully explain exon inclusion, as a few reporters are able to include the RS-exon despite having stronger RP SD, and vice versa. In this regard, RS-exon swap experiments indicate that RS-exon sequences can autonomously instruct their own inclusion. Hence, swapping a cryptic RS-exon in place of an expressed RS-exon results in skipping, whereas opposite effects were observed when an expressed RS-exon replaces a cryptic one. These data hint at the presence of exonic SREs that guide the observed patterns of AS. In general, ESEs are commonly observed within constitutively expressed exons (Wang et al, 2004). This seems likely the case for the expressed *Ubx* RS-exons *m1* and *m2*, which exhibit deep evolutionary conservation across all 51 nt, including wobble positions (Burnette et al, 1999). However, in the case of cryptic RS-exons, it is unclear if these exons contain ESS sequences, or whether the default state for RS-exons (in the absence of SREs) is to activate the RP SD. The latter seems more likely given that cryptic RS-exons (beyond the RP SD) are poorly conserved and are unlikely to contain important regulatory elements.

At this point, we do not know the identity of putative ESE-binding factor(s) that promote RS-exon inclusion. Consistent with previous studies (Hatton et al, 1998), we showed that discrete internal exon sequences beyond the recursive SD are required to include the *Ubx-m1* and *Ubx-m2* RS-exons, which are both ultraconserved. Serine/arginine (SR) proteins are major factors that recognize ESEs, and some *Drosophila* serine/arginine (SR) proteins bind guanosine-rich elements (Bradley et al, 2015; Jeong, 2017). Although we could not implicate obvious candidates from described SR motifs, it remains to be determined if any specific SR proteins are involved in RS-exon splicing. Other studies also implicated splicing factors such as *hrp48*, *virilizer*, and *fl(2)D* in regulation of *Ubx* microexons (Burnette et al, 1999). These have not been subsequently analyzed, but it is perhaps notable that the latter two factors participate not only as more general splicing factors, but also are required in a specific pathway for deposition of m^6^A via the Mettl3/14 complex (Zaccara et al, 2019). Because m^6^A can influence alternative splicing via members of the YTHDC family, perhaps it is worth considering if RNA modifications are germane to this process.

Finally, we demonstrate that pre-removal of the upstream intron segment causes RS-exon skipping. This clearly indicates that SD choice is influenced by the history of previous splicing. This attribute is characteristic of the EJC deposited upstream of exon junctions during splicing (Boehm & Gehring, 2016). We recognize that further evidence is required to provide a direct link between upstream intron splicing and the EJC to regulate Drosophila RS-exon splicing. However, as an analogous function was previously reported in the mammalian system (Blazquez et al, 2018), our experiments are consistent with the notion that the EJC has a conserved function to suppress regenerated splice sites after splicing. Conversely, understanding how cryptic RS-exons (intronic RPs) evade EJC regulation represents a potentially productive future direction.

## Materials and Methods

### Recursive splice site mutants of *kuz* and *Bx*

*Drosophila* RP mutants of *kuz* and *Bx* were generated using CRISPR-Cas9 mutagenesis, as reported previously (Joseph et al, 2018). We used individual gRNA transgenes in the downstream vicinity of the *kuz* and *Bx* RPs. Candidate mutagenized chromosomes from the progeny of sgRNA/Cas9-expressing animals were balanced and analyzed by PCR to identify mutations of interest.

### Constructs and cell culture

The splicing reporter used in this study was reported previously, and contains *kuz* exons (Joseph et al, 2018). For each cloned RS reporter (Fig 3A), we amplified ∼3 kb of intronic sequences containing the RP using PCR. The sequences were cloned into the intronic portion of the *kuz* minigene construct using NotI and EcoRV sites. All RP cloning primers are listed in Table S2. Disruptions of RP SDs were induced using site directed mutagenesis. A similar strategy was used to pre-remove intron segment 1 in RS reporters and to swap RS-exons. Primers used are listed in Table S2.

Table S2 Primer sequences used to clone reporters and assay mRNA processing for recursively spliced substrates.

All transfections in this study were performed using S2-R^+^ cells cultured in Schneider *Drosophila* medium with 10% fetal Bovine serum. Cells were seeded in six-well plates at a density of 1 million/ml and transfected with 200 ng of construct using the Effectene transfection kit (QIAGEN). Cells were harvested following three days of incubation.

### rt-PCR of mRNA and recursive intermediates

To analyze RP SD fly mutants (Figs 1 and 2), we selected homozygous first instar larvae for *kuz* mutants (some of which were lethal), whereas we used homozygous adult female flies for *Bx* mutants (all of which were viable). rt-PCR primers used to analyze animal samples and transfected S2 cell samples are listed in Table S2. S2 cells, mutants and control animals were homogenized and RNA was extracted using the standard Trizol protocol. 5 μg of RNA were treated with Turbo DNase (Ambion) for 45 min before cDNA synthesis using SuperScript III (Life Technologies) with random hexamers. rt-PCRs were performed using AccuPrime Pfx DNA polymerase (Thermo Fisher Scientific) with standard protocol using 32 cycles for mRNA and 34 cycles for intermediates.

### Bioinformatic analyses

We obtained core EJC factor knockdown RNA sequencing datasets from the NCBI Gene Expression Omnibus (GEO) for further analyses (GSE92389). The datasets were reported by the Roignant laboratory (Akhtar et al, 2019). Raw fastq files were mapped to the *Drosophila* reference genome sequence (BDGP Release 5/dm3) using HISAT2 under default settings. Split reads that mapped to neighboring exons with minimum overhangs of 10 nt were used to quantify alternative splicing. For this exercise, gene models corresponding to RS-exon inclusion and skipping were filtered from Ensembl gene annotations. Reads were then assigned and counted as skipped if the split segments mapped exons flanking the RS-exon and as included if the segments mapped a flanking exon and the RS-exon. Relative abundances of skipped and included RS-exons were calculated using “percent spliced in index” (PSI) which is a ratio of RS-exon inclusion-reads count to total spliced-reads count. We applied a minimum total average spliced-reads count filter of nine reads per condition to identify RS-exon genes with robust expression, yielding a set of 10 events (Table S1). Finally, we calculated delta PSIs (EJC KD − *lacZ* KD > 0.2) to evaluate RS events sensitive to core EJC factors.

## Supplementary Material

Reviewer comments
